# Resistance to Degradation and Cellular Distribution are Important Features for the Antitumor Activity of Gomesin

**DOI:** 10.1371/journal.pone.0080924

**Published:** 2013-11-29

**Authors:** Marcus V. Buri, Tatiana M. Domingues, Edgar J. Paredes-Gamero, Rafael L. Casaes-Rodrigues, Elaine Guadelupe Rodrigues, Antonio Miranda

**Affiliations:** 1 Departamento de Biofísica, Universidade Federal de São Paulo, São Paulo, SP, Brazil; 2 Departamento de Bioquímica, Universidade Federal de São Paulo, São Paulo, SP, Brazil; 3 Departamento de Microbiologia, Imunologia e Parasitologia, Universidade Federal de São Paulo, São Paulo, SP, Brazil; Russian Academy of Sciences, Institute for Biological Instrumentation, Russian Federation

## Abstract

Many reports have shown that antimicrobial peptides exhibit anticancer abilities. Gomesin (Gm) exhibits potent cytotoxic activity against cancer cells by a membrane pore formation induced after well-orchestrated intracellular mechanisms. In this report, the replacements of the Cys by Ser or Thr, and the use D-amino acids in the Gm structure were done to investigate the importance of the resistance to degradation of the molecule with its cytotoxicity. [Thr^2,6,11,15^]-Gm, and [Ser^2,6,11,15^]-Gm exhibits low cytotoxicity, and low resistance to degradation, and after 24 h are present in localized area near to the membrane. Conversely, the use of D-amino acids in the analogue [D-Thr^2,6,11,15^]-D-Gm confers resistance to degradation, increases its potency, and maintained this peptide spread in the cytosol similarly to what happens with Gm. Replacements of Cys by Thr and Gln by L- or D-Pro ([D-Thr^2,6,11,15^, Pro^9^]-D-Gm, and [Thr^2,6,11,15^, D-Pro^9^]-Gm), which induced a similar β-hairpin conformation, also increase their resistance to degradation, and cytotoxicity, but after 24 h they are not present spread in the cytosol, exhibiting lower cytotoxicity in comparison to Gm. Additionally, chloroquine, a lysosomal enzyme inhibitor potentiated the effect of the peptides. Furthermore, the binding and internalization of peptides was determined, but a direct correlation among these factors was not observed. However, cholesterol ablation, which increase fluidity of cellular membrane, also increase cytotoxicity and internalization of peptides. β-hairpin spatial conformation, and intracellular localization/target, and the capability of entry are important properties of gomesin cytotoxicity.

## Introduction

Antimicrobial peptides (AMPs) are an evolutionary conserved defense mechanism of animal and plant kingdom [1], [2]. At the last years, AMPs are emerged as a new source of molecules that can be used against different target such as bacteria, fungus, protozoa, and more recently their abilities against tumor cells have been confirmed [3]–[8].

The cationic characteristic of the AMPs has been proposed to be an important feature of its interaction with the outer tumor cell membranes that carries a more net negative charges than non-tumor cells, imparted by negatives molecules such as anionic phospholipids, glycosaminoglicans or negative glycoproteins [7], [9], [10]. In addition, structural characteristics, and biological properties non-identified allow the AMPs to disturb cellular membrane systems being internalized into the cells, this feature seems to be particularly important in their cytotoxic effect [11]–[13]. Several reports have shown different mode of action of these peptides, for instance, AMPs isolated from a wild bee venom such as melectin, lasioglossins, halictines, and macropin induce membrane permeabilization [11], similarly than NK-18 peptide, a mammalian AMP produced by T cells, and natural killer [1]. Conversely, pardaxin, an AMP isolated from secretions of the Red Sea Moses sole, was described to led caspase-dependent, and ROS-mediated apoptosis in fibrosarcoma cell line HT-1080 without membrane permeabilization [9].

Gomesin (Gm) is a β-hairpin AMP isolated from the hemolymph of the Brazilian spider *Ancathoscurria gomesiana* [3], [14], [15]. Its structure includes six alkaline amino acids (1 Lys and 5 Arg), what makes the Gm a cationic peptide (IP calculated  =  9.86), facilitating its interaction with anionic membranes. Moreover, presence of four Cys residues, that forms two disulfide bridges at Cys^2–15^ and Cys^6–11^ positions [3], [14] confers resistance to proteases [16].

Additionally, Gm display cytotoxic activity against tumor cells. The effectiveness of Gm had been shown as a topical agent against B16 melanoma tumor cells [6], neuroblastoma SH-SY5Y, and pheochromocytoma PC12 cells [17]. We recently explore the intracellular mechanisms that promote cell death by Gm in tumor cells demonstrating that the membrane permeabilization induced by Gm is preceded by specific intracellular events such as endoplasmic reticulum disturbance, cytosolic Ca^2+^ increase, followed by an accumulation of Ca^2+^ in organelles, which induces loss of mitochondria potential leading to collapse of mitochondria, which culminates in the disruption of cellular membrane [12], [17].

Due to these diverse results that described the actions of AMPs, membranolytic, and non-membranolytic mechanisms were proposed by different groups [7], [18]. Recently the cytotoxicity ability of β-hairpin AMPs structure in human erythroleukemia K562 cell line was evaluated. The treatment with lower concentrations of AMPs induced controlled cell death mechanisms (e.g. necrosis-like, necroptosis, apoptosis). On the other hand, with higher concentrations of AMPs a direct cell membrane disruption was observed. Among the AMPs tested, gomesin and protegrin, which possess great homology, were the most potent. However, the substitution of Cys by Ser, which leads the AMPs to assume a random conformation due the absence of the disulfide bridges formation, decreased their potency [19]. Those results corroborated with the hypothesis that the activity of Gm has been related with its disulfide bridges that are responsible to its β-hairpin conformation [16], [20].

Despite the investigation of some intracellular mechanism triggered by Gm to induce cell death, its structural characteristics, and biological properties associated with cytotoxic activity in cancer cells remain unclear, similar to others AMPs. In this report, structural modifications were made in the original peptide gomesin in order to better understand the importance of the disulfide bridges, related to resistance to degradation, and antitumor activity in B16 melanoma cell line. We replaced the Cys at positions 2, 6, 11, 15 for Thr, which do not permit the formation of disulfide bridges, changing the β-hairpin structure to a random conformation similar than Ser [20]. Moreover, the peptide with D- or L-Pro at position 9, which induces a β-hairpin-like fold, was also synthesized and tested. Furthermore, we evaluated the interaction of Gm, and its structural analogues on cellular membrane binding, internalization, cellular localization, and resistance to degradation in murine melanoma B16. The modifications in Gm structure allowed us to determine that resistance to degradation, and the ability to entry into the cells are important features related with the cytotoxicity of Gm against B16 melanoma cell line.

## Experimental Procedures

### Peptide synthesis

Peptides were synthesized in-house by the solid-phase methodology on a 4-methylbenzhydrylamine resin (MBHAR) (0.8 mmol/g) according to the *t*-Boc strategy [16]. Full deprotection and cleavage of the peptide from the resin were carried out using anhydrous HF treatment with anisole and dimethyl sulfide (DMS) as scavengers at 0°C for 1.5 h. The formation of disulfide bridges was achieved immediately after HF cleavage and extraction of the crude peptide. The resulting peptide solution was maintained at pH 6.8–7.0 at 10°C during 72 h. Cyclization reactions were monitored by reversed-phase liquid chromatography coupled with an electrospray ionization mass spectrometer (LC/ESI-MS). Lyophilized crude peptides were purified by preparative RP-HPLC on a Jupiter C_18_ column (22.1×250 mm, 300 Å pore size, 15 µm particle size) in two steps. The first step used triethylammonium phosphate (TEAP) pH 2.25 as solvent A and 60% acetonitrile (ACN) in A as solvent B. The second step used 0.1% trifluoroacetic acid (TFA)/H_2_O as solvent A and 60% ACN in A as solvent B. Pure peptides were characterized by amino acid analysis and LC/ESI-MS. Peptides were fluorescently labeled by the addition of rhodamine (Rh) to the Lys side chain by incubating 5 mg of the purified peptides, 2 mg of 5(6)-carboxytetramethylrhodamine N-succinimidyl ester, and 4 µL of N,N-diisopropylethylamine (DIPEA) in 500 µL of N,N-dimethylformamide (DMF) for 2 h at room temperature [21]. Gm and its analogues were biotinylated (Gm-B_12_) by a similar experimental protocol. Briefly, biotin was coupled to the Lys side chain by incubating 8 mg of pure peptide, 2 mg of N-hydroxysuccinimidyl-biotin, and 6 μL of DIPEA in 500 µL of DMF for 2 h at room temperature. The resulting labeled peptides were repurified by preparative RP-HPLC using 0.1% TFA/H_2_O as solvent A and 60% ACN in A as solvent B. Pure peptides were characterized by LC/ESI-MS.

### Cell lines and culture conditions

B16 F10 mouse melanoma cell line was cultured in RPMI medium supplemented with 10% fetal bovine serum (FBS; Gibco, USA), 10 U/ml penicillin and 10 µg/ml streptomycin. Cells were cultured in a humidified incubator containing 2.5% CO_2_ at 37°C.

### Cell viability assay

B16 cells were incubated in 96-well microtiter plates in RPMI medium supplemented with 10% FBS until reach the semi-confluence and then treated with different concentrations of Gm and its analogues for 24 h. To investigate the mechanisms of Gm-induced cell death, B-16 cells were also pre-incubated with 40 µM cytochalasin D (Tocris, USA); 20 µM necrostatin-1 (Tocris, USA), 10 µM Z-VAD (Tocris, USA), 5 mM MβCD (Tocris, USA), and 100 µM chloroquine (Tocris, USA) for 1 h. Cell viability was determined using the standard reduction of the tetrazolium salt 3-(4,5-dimethylthiazol-2-yl)-2,5-diphenyltetrazolium bromide (MTT). The results were expressed relative to control cell viability (100%).

### Cell death by the annexin-V and 7-AAD assay

B16 cells (5×10^5^ cells/ml) were treated with Gm and subsequently harvested, washed with PBS and resuspended in binding buffer (0.01 M HEPES, pH 7.4, 0.14 M NaCl and 2.5 mM CaCl_2_). The suspensions were labeled with annexin-V-APC (An) and 5 µg/ml 7-Amino-actinomycin D (7-AAD) (BD Biosciences, USA) according to the manufacturer’s instructions. After incubation at room temperature for 20 min, cells were analyzed in a FACSCalibur flow cytometer (Becton Dickinson, USA) using CellQuest, and FlowJo 7.6 software. A total of 10,000 events were collected per sample.

### Peptide quantification in membrane and internalized into cells by flow cytometry and confocal microscopy

Cells were treated with the 2 µM biotinylated peptide. Then, the melanoma cells (5×10^5^ for flow cytometry or 5×10^3^ cell seeded for confocal microscopy) were labeled with streptavidin-Alexa 488 (Invitrogen, USA) for 30 min at 4°C and washed with PBS. The cells were fixed with a solution of 2% paraformaldehyde for 30 min, washed with 0.1 M glycin, and permeabilized with 0.01% saponin. Then, the cells were labeled with streptavidin-Alexa 647 (Invitrogen, USA). For flow cytometry, the cells were extracted using PBS with 5 mM EDTA previously to fixation. The quantification of label of external biotynilated peptide, and intracellular peptide were performed by flow cytometry or confocal microscopy. For flow cytometry the cells were excited using argon laser (488 nm), and diode laser (633 nm). The emission was collected in FL-1 and FL-4 channel. A total of 10,000 events were collected per sample. For confocal microscopy, nuclei were stained with DAPI. Confocal microscopy analyses were performed with a confocal laser scanning microscope equipped with a Plan-Apochromat ×63 objective under oil immersion (Zeiss, LSM780). The pinhole device was adjusted to capture fluorescence of one airy unit. The images correspond to single focal plane. Streptavidin-Alexa Fluor 488 were excited using an argon laser (λEx.  =  488 nm, λEm.  =  505–550 nm), streptavidin-Alexa Fluor 647 was excited using a HeNe laser (λEx.  =  633 nm, λEm.  =  640–710 nm) and DAPI was excited using a multiphoton laser (Coherent) (λEx.  =  750 nm, λEm.  =  380–460 nm). Unviable cells were excluded with propidium iodide (PI) label.

### Peptide quantification

The cultured cells were resuspended using pH 5 Tris buffer, and then sonicated. Protein concentration was measured using the RC DC protein assay kit (Bio-Rad, CA, USA) according to the manufacturer’s instructions. The peptides were incubated with this protein solution for 1–14 h, and its stability was evaluated by reversed-phase liquid chromatography coupled with an electrospray ionization mass spectrometer (LC/ESI-MS).

### Statistical analysis

All data represent at least three independent experiments and are expressed as the mean ± standard error of the mean (SEM). Statistical analyses were performed using Student t-test for comparison between two groups and analysis of variance (ANOVA) and Dunnett’s post hoc test for multiple comparisons among groups. A probability (P) value greater than 0.05 was considered significant.

## Results

### Comparison of cytotoxic activity of Gm and analogues

**Figure 1:**
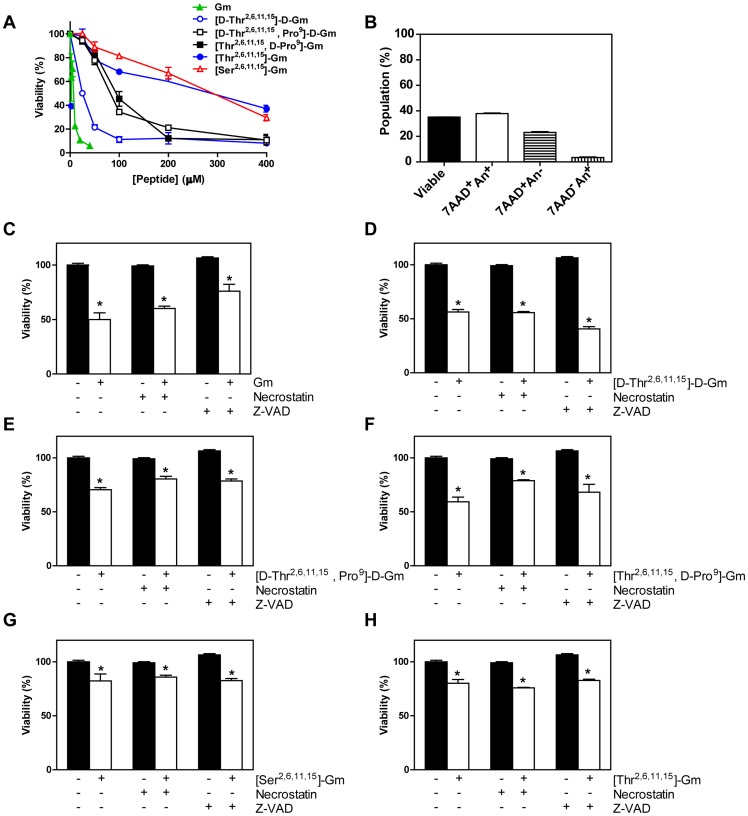
Substitution of some amino acid residues in the Gm structure reduces the cytotoxic ability, but did not modify the cell death mechanism. B16 cells were stimulated by Gm and analogues for 24 h. (**A**) Cytotoxic activities of Gm and its analogues were quantified by the MTT reduction test. (**B**) Cell death type identification caused by Gm was evaluated using annexin-V and 7-AAD assay by flow cytometry using the IC_50_ values. (**C-H**) Apoptosis (Z-VAD) and necroptosis (necrostatin) inhibitors were unable to reduce cell death induced by Gm and analogues. Cells were incubated with inhibitors for 1 h before to stimulation with the peptides (IC_50_ values for each peptide was used) and the viability was assessed by the MTT. Results are the means ± SEM of three independent experiments preformed in duplicate.

Initially the cytotoxic activity of Gm and its analogues were tested in B16 mouse melanoma cell line after 24 h, and then cytotoxicity was evaluated by MTT assay. Substitution of Cys^2–15^/Cys^6–11^ for Thr, Gln^9^ for D- or L-Pro and the use D-amino acids were tested ([D-Thr^2,6,11,15^]-D-Gm, [D-Thr^2,6,11,15^, Pro^9^]-D-Gm, [Thr^2,6,11,15^, D-Pro^9^]-Gm, and [Thr^2,6,11,15^]-Gm), as well as Ser substitution ([Ser^2,6,11,15^]-Gm) that causes a similar effect to Thr substitution, a random conformation, and low antimicrobial activity [6], [16], [20] (Table 1). The cytotoxic results obtained by MTT assay indicated that the rank order of cytotoxic potency was: Gm > [D-Thr^2,6,11,15^]-D-Gm >> [D-Thr^2,6,11,15^, Pro^9^]-D-Gm  =  [Thr^2,6,11,15^, D-Pro^9^]-Gm >> [Thr^2,6,11,15^]-Gm  =  [Ser^2,6,11,15^]-Gm (see Figure 1A). The results of potency and efficacy of the peptides are summarized in Table 2. To identify the type of cell death promoted by Gm and its analogues we used annexin-V, which binds to phosphatidylserine that become exposed to extracellular cell membrane during apoptosis, and 7-AAD, an membrane-impermeant nuclear stain. Gm promotes primarily An^+^7-AAD^+^ label (Figure 1B) that correspond to necrosis-like mechanism by membrane permeabilization observed previously in B16 cells [6], and others tumor cell lines [12], [17]. Similar results were obtained with Gm analogues *(Figure S1)*. To confirm this data we used inhibitor of necroptosis (necrostatin, inhibitor of Rip-1), and apoptosis (Z-Vad, inhibitor of caspases). Both inhibitors were unable to decrease cell death induced by Gm and its analogues (see Figure 1C-H). Suggesting that modification in Gm by substitution of amino acid did not change the mode of cell death triggered by these peptides.

**Table 1:** Primary structure of gomesin and its analogues.

Peptide	Sequence^a^	Calcd mass^b^ (Da)	Obsd mass^b^ (m/z)	Purity^c^ (%)
Gm	Z-C-R-R-L-C-Y-K-Q-R-C-V-T-Y-C-R-G-R-NH_2_	2270,72	2270,5	98
[Ser^2,6,11,15^]-Gm	Z-S-R-R-L-S-Y-K-Q-R-S-V-T-Y-S-R-G-R-NH_2_	2210,49	2210,6	98
[Thr^2,6,11,15^]-Gm	Z-T-R-R-L-T-Y-K-Q-R-T-V-T-Y-T-R-G-R-NH_2_	2265,26	2265,3	99
[D-Thr^2,6,11,15^]-D-Gm	z-t-r-r-l-t-y-k-q-r-t-v-t-y-t-r-g-r-NH_2_	2265,26	2265,5	98
[D-Thr^2,6,11,15^, Pro^9^]-D-Gm	z-t-r-r-l-t-y-k-P-r-t-v-t-y-t-r-g-r-NH_2_	2235,58	2235,7	97
[Thr^2,6,11,15^, D-Pro^9^]-Gm	Z-T-R-R-L-T-Y-K-p-R-T-V-T-Y-T-R-G-R-NH_2_	2235,58	2235,6	98
[Rh-Lys^8^]-Gm	Z-C-R-R-L-C-Y-X-Q-R-C-V-T-Y-C-R-G-R-NH_2_	2683,72	2683,7	99
[Ser^2,6,11,15^; Rh-Lys^8^]-Gm	Z-S-R-R-L-S-Y-X-Q-R-S-V-T-Y-S-R-G-R-NH_2_	2623,49	2623,5	98
[B_12_-Lys^8^]-Gm	Z-C-R-R-L-C-Y-B-Q-R-C-V-T-Y-C-R-G-R-NH_2_	2517,72	2517,9	98
[Ser^2,6,11,15^, B_12_-Lys^8^]-Gm	Z-S-R-R-L-S-Y-B-Q-R-S-V-T-Y-S-R-G-R-NH_2_	2457,49	2457,6	97
[Thr^2,6,11,15^, B_12_-Lys^8^]-Gm	Z-T-R-R-L-T-Y-B-Q-R-T-V-T-Y-T-R-G-R-NH_2_	2512,23	2512,3	98
[D-Thr^2,6,11,15^, B_12_-D-Lys^8^]-D-Gm	z-t-r-r-l-t-y-b-q-r-t-v-t-y-t-r-g-r-NH_2_	2512,23	2512,6	98
[D-Thr^2,6,11,15^, B_12_-D-Lys^8^, Pro^9^]-D-Gm	z-t-r-r-l-t-y-b-P-r-t-v-t-y-t-r-g-r-NH_2_	2482,58	2482,6	99
[Thr^2,6,11,15^, B_12_-Lys^8^, D-Pro^9^]-Gm	Z-T-R-R-L-T-Y-B-p-R-T-V-T-Y-T-R-G-R-NH_2_	2482,58	2482,7	97

a Z  =  pyroglutamic acid, lowercase letters denote D-amino acids, X  =  Rh-Lys, Rh =  rhodamin; B  =  B_12_-Lys, B_12_ =  biotin. ^b^The observed m/z of the unresolved peak was compared with the calculated [M + H]^+^ average mass in Da.^c^Percent purity as determined by HPLC analysis performed on a Waters Nova-Pak C_18_ (2,1×150 mm, 60 Å, 3,5 µm); UV detection at 214 nm; 0.4 mL/min flow rate; [A]  =  0.1% TFA in H_2_O and [B]  =  0.1% TFA in 60% MeCN/H2O; gradient  =  5–95%B in 30 min.

**Table 2:** Potencies and efficacies of gomesin and its analogues.

Peptide	IC_50_ (µM)	* Max. External Fluorescence (AU)	* Max. Internal Fluorescence (AU)	# Peptide Degradation (%)
Gomesin	7	30±8	603±79	0±0
[D-Thr^2,6,11,15^]-D-Gm	25	43±6	602±12	12±2
[D-Thr^2,6,11,15^, Pro^9^]-D-Gm	75	71±4	2751±190	15±2
[Thr^2,6,11,15^, D-Pro^9^]-Gm	90	38±6	1165±27	16±1
[Ser^2,6,11,15^]-Gm	>200	37±3	305±65	47±5
[Thr^2,6,11,15^]-Gm	>200	17.5± 7	753±175	43±4

* To quantify internal or external amount fluorescence biotinylated peptides were used.

# Starting peptide concentration (10^−4^ M) was considered as 100%.

### Potentialization of cytotoxic Gm effect by chloroquine

**Figure 2:**
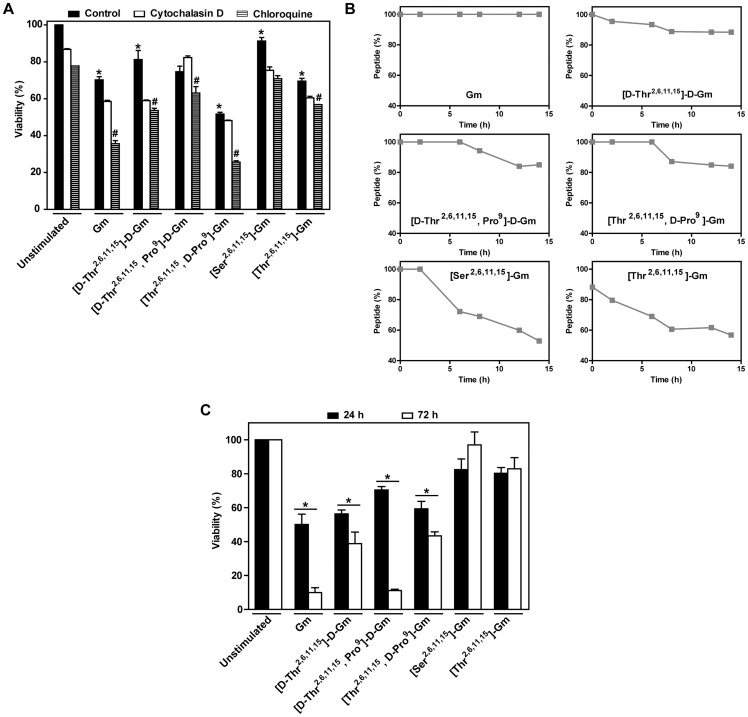
Lysosomal enzymes participate in the cytotoxic reduction activities of the Gm analogues. (**A, C and D**) Cell viability was assessed by the MTT reduction test. (**A**) Cells were incubated with cytoskeletal inhibitor (cytochalasin D), and lysosomal inhibitor (chloroquine) for 1 h before to stimulation by Gm, and its analogues for 24 h. Chloroquine potentiated the cytotoxicity of peptides. (**B**) Since chloroquine was able to potentiate the cytotoxicity of the peptides, resistance to degradation of lysosomal enzymes was evaluated by LC/ESI-MS. Peptides were incubated at 37°C for different times. (**C**) Cells were incubated with the IC_50_ concentration for each peptide. Cytotoxic activities after 24 and 72 h of the peptide incubation were compared.

As necroptosis and apoptosis inhibitors were unable to block the cell death promoted by Gm and analogues other inhibitors were employed. The participation of free radicals in the cell death induced by gomesin and its analogues were evaluated by the use of N-Acetyl-Cysteine (NAC), a free radical scavenger, which was unable to significantly inhibit cell death induced by the peptides (Data not shown). The use of cytochalasin D, a potent disruptor of actin filament function that blocks endocytosis mechanisms, were ineffective (Figure 2A). However, chloroquine, which concentrates in lysosomes, and raises their medium pH disrupting the function of lysosomal enzymes, potentiate the effects of Gm and its analogues, suggesting that the effects of peptides could be related with degradation by lysosome enzymes. Therefore, the resistance to lysosomal enzyme degradation of Gm and analogues were quantified. We observed a direct correlation between resistance of degradation, and cytotoxic activity. Gm shows great stability to lysosomal enzymes, and was the most potent peptide, whereas [Thr^2,6,11,15^]-Gm and [Ser^2,6,11,15^]-Gm that are the most sensitive peptides to degradation exhibit lower activity (Figure 2B). As expected [D-Thr^2,6,11,15^]-D-Gm, and [D-Thr^2,6,11,15^, Pro^9^]-D-Gm were little affected by lysosomal enzymes, and unexpectedly [Thr^2,6,11,15^, D-Pro^9^]-Gm was also stable to enzymatic degradation may be due a structural fold caused by introduction of the D-Pro residue (Figure 2B). Since Gm and some analogues were little degraded, we decide to incubate the cells with the peptides, and observed cytotoxic effect after 72 h. The peptides incubation after 72 h caused an increase in the cytotoxicity of the ones that are more resistant to degradation, whereas the activity of the less resistant peptides ([Ser^2,6,11,15^]-Gm an [Thr^2,6,11,15^]-Gm) was not alter (Figure 2C).

### Quantification of binding to cell membrane and internalization of Gm and its analogues

**Figure 3:**
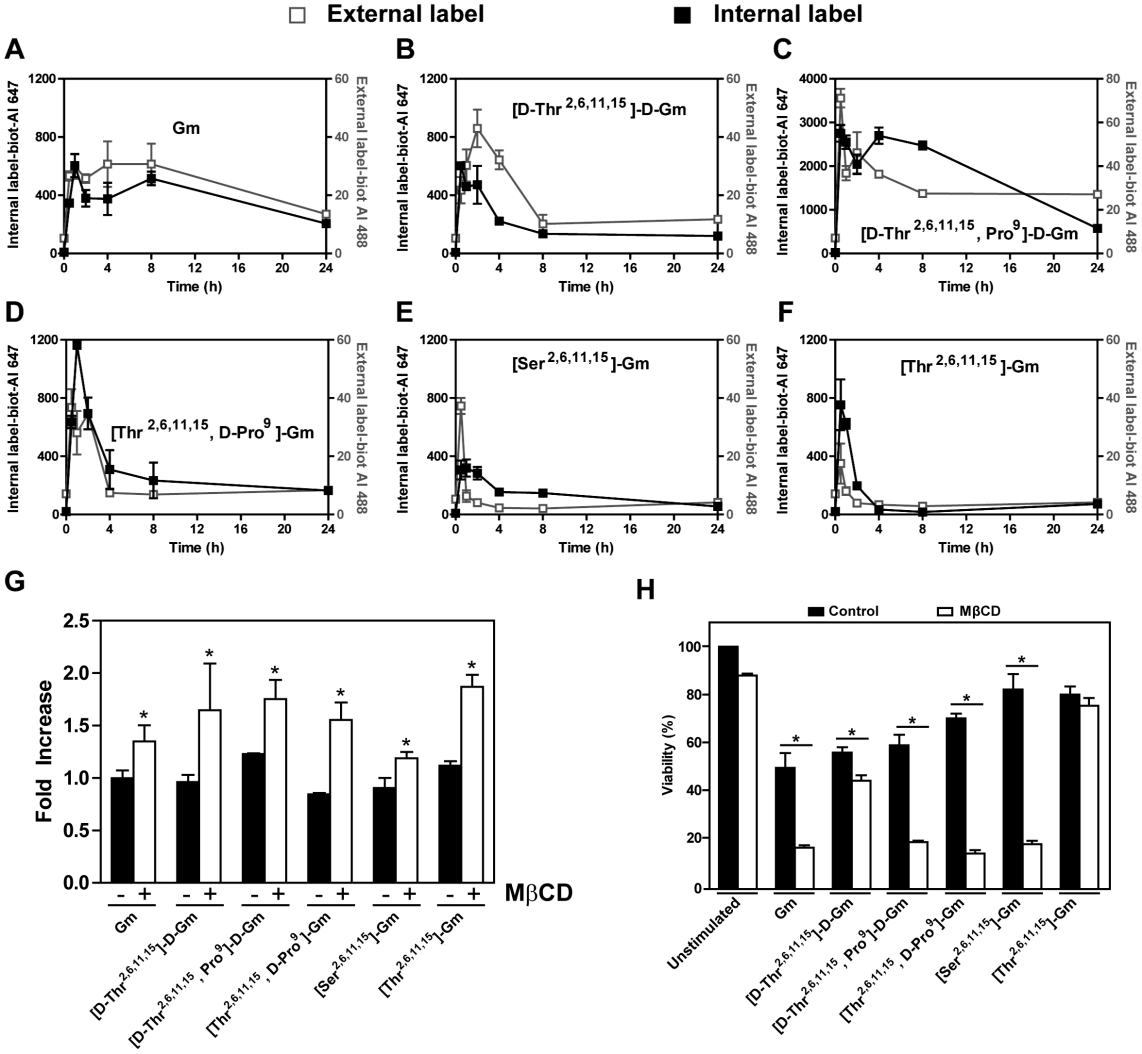
Cytotoxic effect of antimicrobial peptides is related to the entry of peptides but not with the binding to the cell membrane. B16 cells were incubated with 2 µM of the biotin labeled peptides for different times. Quantification of the peptides were done by flow cytometry. External membrane peptides were labeled with streptavidin-Alexa Fluor 488 conjugated. Internal membrane peptides were labeled with streptavidin-Alexa Fluor 647 conjugated after fixation, and permeabilization. Unviable cells were excluded using PI stain. External and internal quantifications of (**A**) Gm, (**B**) [D-Thr^2,6,11,15^]-D-Gm, (**C**) [D-Thr^2,6,11,15^, Pro^9^]-D-Gm, (**D**) [Thr^2,6,11,15^, D-Pro^9^]-Gm, (**E**) [Thr^2,6,11,15^]-Gm, and (**F**) [Ser^2,6,11,15^]-Gm are shown. (**G-H**) To decrease cholesterol of the membrane, the cells were incubated with MβCD for 1 h. (**G**) This treatment enhance the entry of peptides, (**H**) and potentiates the cytotoxic effect of most of them. Results are the means ± SEM of three independent experiments preformed in duplicate.

We quantified the Gm and analogues binding to the cellular membrane, and their internalization using their biotinylated counterpart. The cells were incubated at different times using a low concentration of the peptides (2 µM) that does not induces membrane permeabilization. Unviable cells were excluded by PI label *(Figure S2)*. External peptides labeled with streptavidin Alexa Fluor 488 were identified for the quantification of the green fluorescence (FL1 channel), and internal peptides labeled with streptavidin Alexa Fluor 647 were identified for the quantification of the deep red fluorescence (FL4 channel), after permeabilization of cellular membrane, by Flow cytometry. Quantification of external and internal label showed a rapid binding of the peptides with a peak at 2–4 h for all peptides (Figure 3A-F). [Thr^2,6,11,15^, D-Pro^9^]-Gm exhibits the highest binding among the peptides tested. Gm and [D-Thr^2,6,11,15^]-D-Gm, the most cytotoxic peptides, presents a medium value of binding. [Thr^2,6,11,15^]-Gm and [Ser^2,6,11,15^]-Gm, which possess the lower cytotoxic activity, were the peptides that less binding to cellular membrane (Figure 3A-F). The temporal quantification of internalized peptides shows that peptides follow a similar temporal behavior. It is possible to observe the internal presence of Gm, [D-Thr^2,6,11,15^]-D-Gm, [D-Thr^2,6,11,15^, Pro^9^]-D-Gm, and [Thr^2,6,11,15^, D-Pro^9^]-Gm after 24 h. On the other hand, the peptides that present lower cytotoxicity were not retained after 24 h (Figure 3A-F). However, a direct relation between binding, peptide entrance, and cytotoxicity effect was not full observed, the entrance into cells seem to be important to the action of several AMPs [12]. To verify the participation of the entry of peptide with cytotoxicity cholesterol level cholesterol level was reduced incubating the cells with MβCD for 1 h, which increase the membrane fluidity [22]. As expected, the treatment of the cells with MβCD increases the entry of peptide (Figure 3G) and increases the cytotoxicity of the peptides, except the analogue [Thr^2,6,11,15^]-Gm (Figure 3H).

### Cellular localization of Gm and its analogues

**Figure 4:**
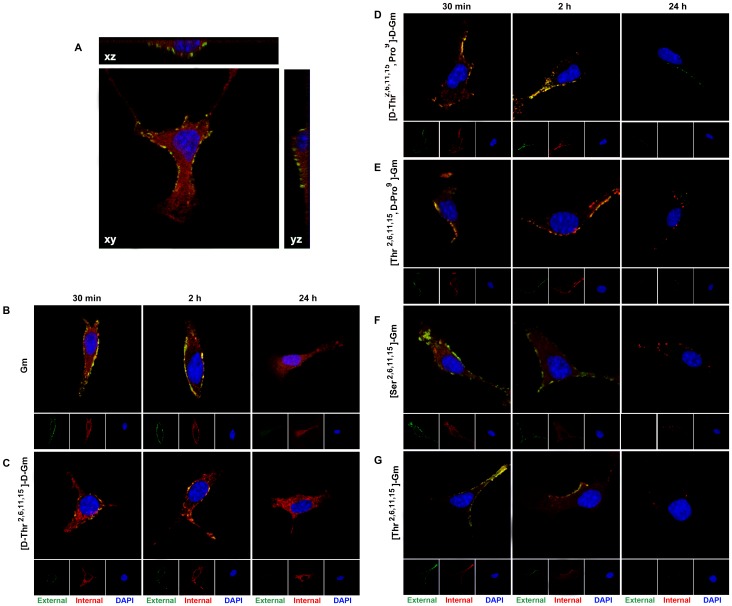
External or internal localization of Gm and analogues. B16 cells were incubated with 2 µM of the biotin labeled peptides for different times. Images correspond to a single focal plane performed by confocal microscopy. External membrane peptides were labeled with streptavidin-Alexa Fluor 488 conjugated while internal membrane peptides were labeled with streptavidin-Alexa Fluor 647 conjugated. Unviable cells were excluded using PI stain. Nuclei were stain with DAPI. (**A**) Typical image of labeled Gm is showed. The images XY, XZ and YZ correspond to a single plane. Typical images obtained with (**B**) Gm, (**C**) [D-Thr^2,6,11,15^]-D-Gm, (**D**) [D-Thr^2,6,11,15^, Pro^9^]-D-Gm, (**E**) [Thr^2,6,11,15^, D-Pro^9^]-Gm, (**F**) [Ser^2,6,11,15^]-Gm and (**G**) [Thr^2,6,11,15^]-Gm are shown.

In order to visualize the cellular localization of Gm and analogues we used them labeled with biotin, and the localization of the peptides were performed using confocal microscopy. The images obtained by confocal microscopy were obtained of a single focal plane (XY) except Figure 4A. The green color corresponds to external label of peptides, and red label correspond to internal label after permeabilization of cellular membrane. A representative image obtained with a cell incubated with Gm is shown in Figure 4A. It is possible to observe the peptide presence outside, and inside of cell in XZ and YZ images. The samples were incubated with the biotilynated peptides for 30 min, 2 h, and 24 h. External peptides can be observed at 30 min and 2 h of stimulus, and it is also possible to observe several co-localized pixels (yellow) in the figures due to the accumulation of red label in membrane area (Figure 4). The co-localization means that part of the peptides was on external surface of the cellular membrane, and other part was internalized, but retained in the cell inside. High levels of Gm and [D-Thr^2,6,11,15^]-D-Gm, the most cytotoxic peptides, in the cytoplasm are observed. (Figure 4B and C). Interestingly, different patterns of peptide distribution could be observed after 24 h. There are no evidences of external labeled Gm or [D-Thr^2,6,11,15^]-D-Gm after 24 h, but both peptides remain spread in the cytoplasm (Figure 4B and C). [D-Thr^2,6,11,15^, Pro^9^]-D-Gm still remains present after 24 h in the exterior cellular membrane (Figure 4D). Conversely, [Thr^2,6,11,15^, D-Pro^9^]-Gm, [Thr^2,6,11,15^]-Gm, and [Ser^2,6,11,15^]-Gm are only present near to membrane in the form of vesicles (Figure 4E-G).

## Discussion

Although several reports have described antitumor activity of AMPs, few reports have investigated what are the key characteristics required to promote this effect. In this study, important properties of AMPs that could be related with their cytotoxic activity against B16 melanoma lineage were identified. Therefore, the binding ability to cellular membrane, the internalization of the peptide, its resistance to degradation, and cellular localization were evaluated, and compared between Gm and its analogues. Among these properties, resistance to degradation and entry of peptides into the cells showed to be the most important characteristics associated with Gm cytotoxicity.

Gm is a β-hairpin peptide folded by two disulfide bridges in Cys^2–15^/Cys^6–11^ related with degradation resistance [16], [20]. The replacement of the Cys residues by Ser or Thr caused a decrease of the cytotoxic activity as can be observed with the analogues ([Thr^2,6,11,15^]-Gm), and ([Ser^2,6,11,15^]-Gm) (Figure 1A). This amino acids replacements causes a severe conformation change (from β-hairpin to a random conformation), and consequently an abrupt reduction in their antimicrobial [6], [16], [20], and cytotoxic activities (Figure 1A). In addition, was also observed that both analogues, [Ser^2,6,11,15^] and [Thr^2,6,11,15^]-Gm, have low degradation resistance when incubated with lysosomal enzymes (Figure 2B), or even in blood serum [16], [20]. In order to increase the resistance to enzymatic degradation, the analogue [D-Thr^2,6,11,15^]-D-Gm was synthesized, and showed to be unrecognized by the enzymes (Figure 2B). Moreover, the introduction of D-Pro or L-Pro residue in the Gm or in D-Gm sequences were also evaluated. These substitutions maintained a β-hairpin fold [20], increasing the resistance to degradation by lysosomal enzymes (Figure 2B), and blood serum [16], [20].

### Effect of low and high peptide concentration and peptide binding ability to cell membrane

Gm was the most potent peptide to induce cell death with an IC_50_ around 7 µM. As previously report, β-hairpin AMPs can activate several intracellular mechanisms to trigger cell death mechanisms such as apoptosis, necroptosis or necrosis-like with low concentrations (below IC_50_), but with higher concentrations (above IC_50_) AMPs promote direct perturbation in the cellular membrane [13]. Gm and its analogues induce cell death with necrosis-like features at IC_50_, and apoptosis (Z-VAD) or necroptosis (Necrostatin) inhibitors (Figure 1) did not inhibit their actions. These mechanism seem to be dependent of cell type [6], [8],[12],[19]. Although, to cell death occur the first step is the AMPs interaction with the cell membrane. Biotin-labeled peptides were tested to quantify the outside membrane interaction of the peptides. However, the order of binding ability of the peptides did not directly correspond to their cytotoxicity ([D-Thr^2,6,11,15^, Pro^9^]-D-Gm > [Thr^2,6,11,15^, D-Pro^9^]-Gm > Gm  =  [D-Thr^2,6,11,15^]-D-Gm > [Thr^2,6,11,15^]-Gm > [Ser^2,6,11,15^]-Gm). However, some differences in the shape of the curves of the peptides binding to cellular membrane over the time were also noticed (Figure 3).

### Peptides internalization mechanism

After peptide binding to the cell membrane, internalization by direct translocation or endocytosis mechanisms could happen. However, until now the main mechanism associated with AMPs internalization is not well clarified. Some reports have shown that internalization of AMPs occur by endocytosis mechanisms [23], because it can be blocked by inhibitors of macropinocytosis, such as amiloride, or cytochalasin D, an F-actin elongation inhibitor [24]. Nevertheless, this mechanism cannot be generalized for all AMPs [25]. It is probably that the peptide internalization, by the classic AMP action model (carpet, toroidal pore, barrel stable pore or inverted micelle), and endocytosis-mediated mechanisms (macropinocytosis, clathrin-dependent, caveolin-dependent or clathrin/caveolin independent) occur simultaneously. Intrinsic feature of AMP use, its concentration are important to action. Type of cell studied is also relevant, since different mode of action with the same AMP occur in different cell types [6], [13], [17], [26], [27]. Apparently peptides quantity present inside of cells ([D-Thr^2,6,11,15^, Pro^9^]-D-Gm > [D-Thr^2,6,11,15^]-D-Gm  =  [Thr^2,6,11,15^, D-Pro^9^]-Gm  =  [Ser^2,6,11,15^]-Gm  =  Gm > [Thr^2,6,11,15^]-Gm) did not have a directly correspondence to the cytotoxic potency of the compounds (Figure 3A-F). Nevertheless, the increase of membrane fluidity that increase entrance of peptides arise the potency of most them (Figure 3G-H). Thus, the entry of peptide is an important feature to cytotoxic activity.

### Effect of the peptide degradation resistance

Among the peptides tested in this study, Gm and its linear analogue [D-Thr^2,6,11,15^]-D-Gm were the most potent compounds. However, [D-Thr^2,6,11,15^]-D-Gm showed to be 3.5-fold less potent than Gm. Some differences were noticed between these two peptides. For instance, despite of the fact that Gm and [D-Thr^2,6,11,15^]-D-Gm binding to external cell membrane and are present at similar amount into the cell, faster decrease of intracellular amount can be observed for [D-Thr^2,6,11,15^]-D-Gm (Figure 4A and B). Rapid reduction of the peptide amount into the cell could occur by degradation or extrusion of the peptide from the cell. Lysosomal enzyme inhibition with chloroquine potentiates the response of all peptides utilized in this study, suggesting the participation of lysosomal enzymes in degradation of the internalized peptides. Lysosomal enzymes reduce poorly the effect of [D-Thr^2,6,11,15^]-D-Gm (Figure 2B), but this could be related to the decrease potency of this compound. Interestingly, both peptides, Gm and [D-Thr^2,6,11,15^]-D-Gm, are also rapid translocated into the cytoplasm of B16 cells, and are retained spread in intracellular content after 24 h (Figure 4A and C). However, further investigations are needed to identify the enzymes involved in the AMP degradation, which might be useful to generate new resident compounds.

Different internal distributions of Gm and analogues were observed after internalization of peptides that can be related with enzyme resistance ability (Figure 4). After 30 min, and 2 h of incubation all peptides, with the exception of [Ser^2,6,11,15^]-Gm, exhibit a great co-localization in the outer, and inner sides of the cell membrane. The co-localization of the external and internal labeled peptide means that certain amounts of molecules are present on the cell surface, and the other is present inside the cell. This is due to the resolution limit of the confocal microscopy (∼200 nm) and the thickness of cellular membrane (∼20 nm) [28]. Furthermore, the AMPs may have different ways to cross the cell membrane, and also present different release capabilities into the cytoplasm, these two phenomenas could be very important properties, and could be related with the spatial conformation when the AMPs are crossing the lipid bilayer. Although, only Gm and [D-Thr^2,6,11,15^]-D-Gm are present in high amount in the cytoplasmatic medium (Figure 4B and C). Interestingly, after 24 h Gm and [D-Thr^2,6,11,15^]-D-Gm still remain spread in the cytoplasm, while the other peptides apparently are inside of vesicles near to the membrane or inserted in the cellular membrane (Figure 4B-G). The former observation is in agreement with several reports of the literature that described that the penetration, and distribution of the AMP did not happen in the same way; in fact some AMPs are present in the cytoplasm, and others are in vesicles depending on the peptide involved [24], [25], [29]. Apparently, Gm and [D-Thr^2,6,11,15^]-D-Gm are resistant to cell extrusion mechanism, while the other peptides studied are not, probably associated with great enzyme resistance feature. This also can explain the potentiation of cell death after 72 h of the peptides with most cytotoxic activity.

## Conclusions

Summarize, we showed the importance of the resistance to degradation in the cytotoxic activity of Gm and its analogues probably associated with intracellular distribution, and extrusion. In addition, we also noticed that the entry capability is also related with the resistance to degradation and peptide conformation.

## Supporting Information

Figure S1 **Cell death type identification caused by Gm and its analogues was evaluated using annexin-V and 7-AAD assay by flow cytometry using the IC_50_ values.** Results are the means ± SEM of three independent experiments preformed in duplicate.
(TIF)

Figure S2 **Secondary control sample. (A) Control cells labeled with streptavidin-Alexa Fluor 488 and streptavidin-Alexa Fluor 647 in absence of biotynilated peptides. (B) Unviable cells were excluded using PI stain.** A representative cell is showed. Nuclei were stain with DAPI. Confocal microscopy images were performed in a LSM780 (Zeiss) system.
(TIF)
