# Supplementary figures and images for: Resistance to Degradation and Cellular Distribution are Important Features for the Antitumor Activity of Gomesin
Source: PLoS One. 2013 Nov 29;8(11):e80924. doi: 10.1371/journal.pone.0080924 (PMC3843672; doi:10.1371/journal.pone.0080924)

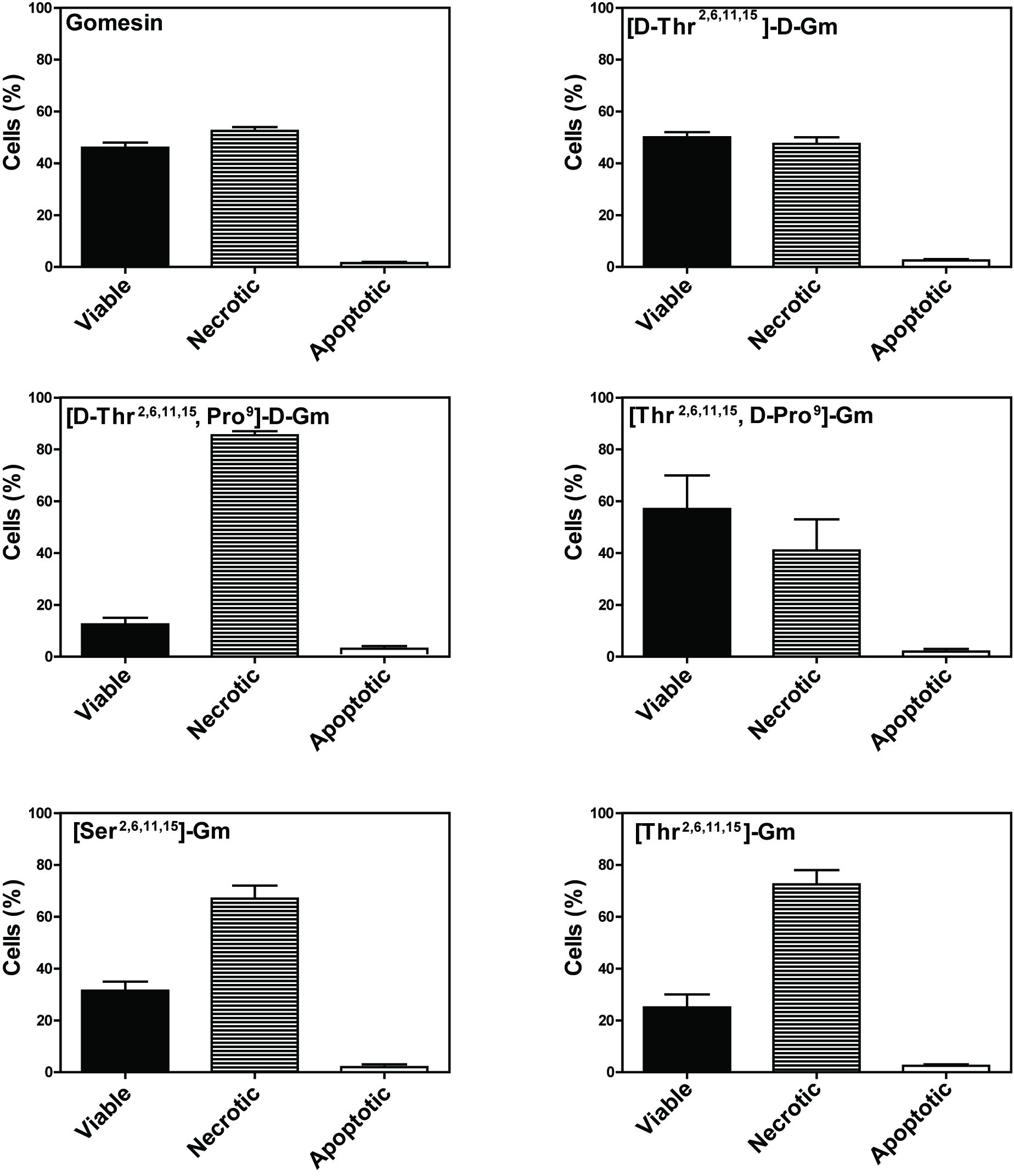

Supplement: Figure S1 — Cell death type identification caused by Gm and its analogues was evaluated using annexin-V and 7-AAD assay by flow cytometry using the IC50 values. Results are the means ± SEM of three independent experiments preformed in duplicate. (TIF) [file pone.0080924.s001.tif]

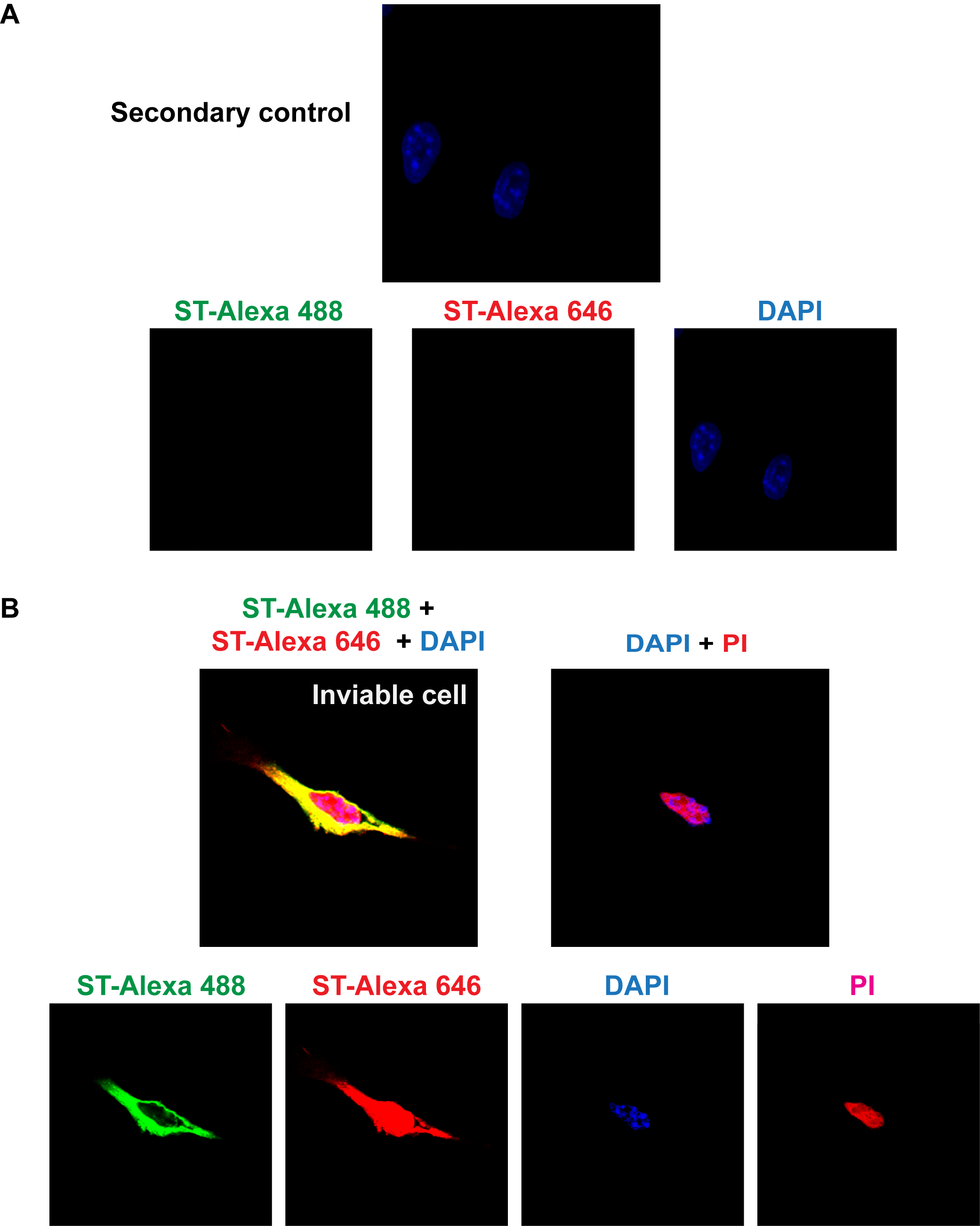

Supplement: Figure S2 — Secondary control sample. (A) Control cells labeled with streptavidin-Alexa Fluor 488 and streptavidin-Alexa Fluor 647 in absence of biotynilated peptides. (B) Unviable cells were excluded using PI stain. A representative cell is showed. Nuclei were stain with DAPI. Confocal microscopy images were performed in a LSM780 (Zeiss) system. (TIF) [file pone.0080924.s002.tif]
